# Multihormonal pituitary adenoma concomitant with Pit-1 and Tpit lineage cells causing acromegaly associated with subclinical Cushing’s disease: a case report

**DOI:** 10.1186/s12902-017-0203-5

**Published:** 2017-09-02

**Authors:** Tomoko Takiguchi, Hisashi Koide, Hidekazu Nagano, Akitoshi Nakayama, Masanori Fujimoto, Ai Tamura, Eri Komai, Akina Shiga, Takashi Kono, Seiichiro Higuchi, Ikki Sakuma, Naoko Hashimoto, Sawako Suzuki, Yui Miyabayashi, Norio Ishiwatari, Kentaro Horiguchi, Yukio Nakatani, Koutaro Yokote, Tomoaki Tanaka

**Affiliations:** 1 Department of Clinical Cell Biology and Medicine, Chiba University Graduate School of Medicine, Chiba, 260-8670 Japan; 2 Department of Diabetes, Endocrinology and Metabolism, Chiba University Hospital, Chiba, 260-8670 Japan; 3 Department of Molecular Diagnosis, Chiba University Graduate School of Medicine, Chiba, 260-8670 Japan; 4 Department of Neurological Surgery, Chiba University Hospital, Chiba, 260-8670 Japan; 5 Department of Pathology, Chiba University Hospital, Chiba, 260-8670 Japan

**Keywords:** Case report, GHoma, Subclinical Cushing’s disease, Pituitary adenoma, Transcriptional factor

## Abstract

**Background:**

A functional pituitary adenoma can produce multiple anterior-pituitary hormones, such as growth hormone (GH) -producing adenomas (GHoma) with prolactin or thyrotropin stimulating hormone production in the same lineage. However, it is very rare that acromegaly shows subclinical Cushing’s disease (SCD) beyond the lineage. Here we describe the involvement of intratumoral coexistence with 2 types of hormone-producing cells associated with different lineage in acromegaly concomitant with SCD.

**Case presentation:**

In our study, we performed clinical evaluation of the patient showing acromegaly with SCD. To elucidate the mechanisms of this pathology, we analyzed immunohistochemistry and gene expression of anterior-pituitary hormones and transcriptional factors in the resected pituitary tumor. On immunohistochemical staining, most of the tumor cells were strongly stained for GH antibody, while some cells were strongly positive for adrenocorticotropic hormone (ACTH). Gene expression analysis of a transsphenoidal surgery sample of the pituitary gland revealed that ACTH-related genes, such as *POMC, Tpit,* and *NeuroD1* mRNA, had higher expression in the tumor tissue than the nonfunctional adenoma but lower expression compared to an adenoma of typical Cushing’s disease. Further, double-labeling detection methods with a fluorescent stain for ACTH and GH demonstrated the coexistence of ACTH-positive cells (GH-negative) among the GH-positive cells in the tumor. Additionally, Pit-1 expression was reduced in the ACTH-positive cells from tumor tissue primary culture.

**Conclusion:**

Here we described a case of a pituitary tumor diagnosed with acromegaly associated with SCD. We performed quantitative-expression analyses of transcriptional factors of the tumor tissue and immunohistochemistry analysis of tumor-derived primary culture cells, which suggested that the multihormonal pituitary adenoma concomitant with Pit-1 and Tpit lineage cells caused acromegaly associated with SCD.

## Background

A functional pituitary adenoma can occasionally produce multiple anterior-pituitary hormones. The expression of lineage-specific transcription and coupling factors for pituitary differentiation plays an important role in the pathologies of functional acquisition for these tumors. Developmentally, determination of anterior-pituitary cell type lineage results from a temporally regulated cascade of homeodomain transcriptional factors. Pit-1 is a transcriptional factor that determines development and appropriate temporal and spatial expression of cells committed to growth hormone (GH), prolactin (PRL), thyroid-stimulating hormone (TSH), and gonadotropin-releasing hormone (GHRH) receptor expression [1]. Corticotroph cell commitment is independent for Pit-1-determined lineages, and Tpit is required for pro-opiomelanocortin (POMC) expression [2]. Accordingly, GH-producing adenomas (GHoma) are often accompanied by PRL or TSH production as the same lineage [3].

However, it is very rare that acromegaly shows subclinical Cushing’s disease (SCD) beyond the lineage, but some cases were reported. The involvement of intratumoral coexistence with 2 types of hormone-producing cells associated with different lineage has been proposed to explain the occurrence of clinical SCD with a GH-producing pituitary tumor [4–6]. On the other hand, involvement of aberrant expression of lineage-specific transcription factors, false positive due to a hyperglycemic state, and effect of GH/insulin-like growth factor 1 (IGF-1) on cortisol metabolism-related enzyme 11β-hydroxysteroid dehydrogenase (11-βHSD) have been reported [7–9].

Here we report a 45-year-old man diagnosed with acromegaly associated with SCD by endocrinological examination. Transsphenoidal surgery was performed and quantitative-expression analyses of transcriptional factors of the tumor tissue, and immunohistochemistry analysis of tumor-derived primary culture cells and the tumor tissue revealed that the tumor consisted of multihormonal-pituitary adenoma cells concomitant with Pit-1 and Tpit lineage cells causing acromegaly associated with SCD.

## Case presentation

A 45-year-old Japanese man was diagnosed with type 2 diabetes mellitus (DM) 12 years before and was prescribed anti-diabetic drugs. Because glycemic control gradually worsened, insulin treatment was started 6 years before. He noticed symptoms such as difficulty taking off his ring and having a larger shoe size at that time. He visited a local doctor because he experienced sudden headache and vertigo. Because a pituitary tumor was identified on brain magnetic resonance imaging (MRI), the patient was referred to our hospital for further examination and treatment. In his head and neck region, typical acromegalic features were observed, such as frontal skull bossing, cranial ridges, enlargement of the nose and lips, and macroglossia. However, typical cushingoid features, such as moon face, buffalo hump, central obesity, and purple striae, were not observed.

Biochemical analyses were performed at admission. Hemoglobin A1c (NGSP) level was 11.0% and poor glycemic control was noted (fasting/2 h after meal: blood glucose, 161/298 mg/dL). An increase in GH and IGF-1 basal plasma levels was observed (78.5 ng/mL and 525 ng/mL (+5.8 SD), respectively). Furthermore, adrenocorticotropic hormone (ACTH) level was above the reference range. The ACTH, cortisol, and dehydroepiandrosterone sulfate levels were 47.8 pg/mL, 13.6 μg/dL, and 247 μg/dL, respectively. Other thyroid dysfunction or gonadal axis abnormalities were not observed (Table 1).

**Table 1:** Endocrine data before and after surgery

	Normal range	Pre-operative	Post-operative
8:00 ACTH (pg/ml)	<46.0	47.6	59.6
23:00 ACTH (pg/ml)		22.6	18.2
8:00 Cortisol (μg/dl)	5.0–25.0	13.6	9.6
23:00 Cortisol (μg/dl)		4.8	2.5
Dehydroepiandrosterone sulfate (μg/dl)	70–495	247	163
GH (ng/ml)	0.04–3.60	78.5	16.9
Insulin like growth factor-1 (ng/ml)	90–250	525	488
LH (μg/dl)	1.14–8.75	3.46	3.03
FSH (pg/ml)	1.37–13.58	8.29	7.36
PRL (ng/ml)	3.46–26.53	10.17	7.74
TSH (μIU/ml)	0.350–4.940	0.664	0.492
Free triiodothyronine (ng/dl)	1.71–3.71	1.49	1.55
Free thyroxine (pg/ml)	0.70–1.48	1.11	1.18
Arginine vasopression	<3.8	ND	2.6
C-peptide immunoreactivity (ng/ml)	0.67–2.48	0.50	0.80
Urinary free cortisol (μg/day)	11.2–80.3	114.3	130.5
0.5 mg-Dexamethasone cortisol	<5.0	3.8	5.6
8 mg-Dexamethasone cortisol		<1.0	<1.0

*Abbreviations: ACTH* adrenocorticotropic hormone, *GH* growth hormone, *LH* luteinizing hormone, *FSH* follicle-stimulating hormone, *PRL* prolactin, *TSH* thyroid-stimulating hormone, *GH* growth hormone

After adequate glycemic control (fasting/2 h after meal: blood glucose, 87/119 mg/dL), a 75-g oral glucose tolerance test (OGTT) was performed, but GH secretion was not suppressed appropriately. In addition, as shown in Table 1, to evaluate the hypothalamic–pituitary–adrenal (HPA) axis we performed the following analyses. The patient showed no circadian variation in ACTH or cortisol secretion. The urine cortisol was 114.3 μg/day, indicating the increased cortisol secretion. In a low-dose dexamethasone (DEX) loading test (0.5 mg) as described previously [10], ACTH and cortisol secretion was not suppressed; however, ACTH and cortisol secretion was suppressed with high-dose (8 mg) DEX loading (Table 1). In response to an intravenous administration of corticotrophin-releasing hormone loading test, ACTH was increased approximately 2.8 times (vor: 38.9 pg/ml, 15 min: 113.0 pg/ml) and cortisol responded (vor: 15.0 μg/dL, 30 min 21.0 μg/dL). Good glycemic control was observed during evaluation of the HPA axis.

**Fig. 1:**
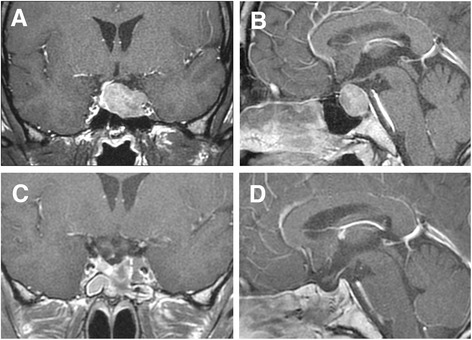
Magnetic resonance imaging (MRI) of the brain. Coronal (**a**) and sagittal (**b**) pre-operative gadolinium-enhanced T1-weighted MRI shows the presence of a macroadenoma. Coronal (**c**) and sagittal (**d**) post-operative MRI shows the presence of small residual tumors

MRI of the brain: a tumor 31 × 20 × 22 mm in diameter was found spanning the inside of the sella turcica to the upper part of the sella (Fig. 1a and b). The pituitary stalk was displaced to the right side (slightly upward) and was adjacent to some parts of the optic chiasm on the right side. The tumor extended into the left cavernous sinus and engulfed the left internal carotid artery indicating grade 4 of the Knosp classification.

The patient was diagnosed with acromegaly according to these clinical and laboratory findings. The abnormal ACTH-dependent cortisol secretion was considered to be associated with SCD. Long-acting repeatable formulation of octreotide (Sandostatin® LAR Depot) was administered monthly for 3 months before the surgery. After an approximately 23% tumor reduction effect was obtained with octreotide, transsphenoidal surgery was performed in the neurosurgery department. During the surgery, a white soft tumor was found in the inner left side of the sella turcica. Infiltration of the tumor to the cavernous sinus was observed, but gross resection was attempted to the extent possible (Fig. 1c and d).

**Fig. 2:**
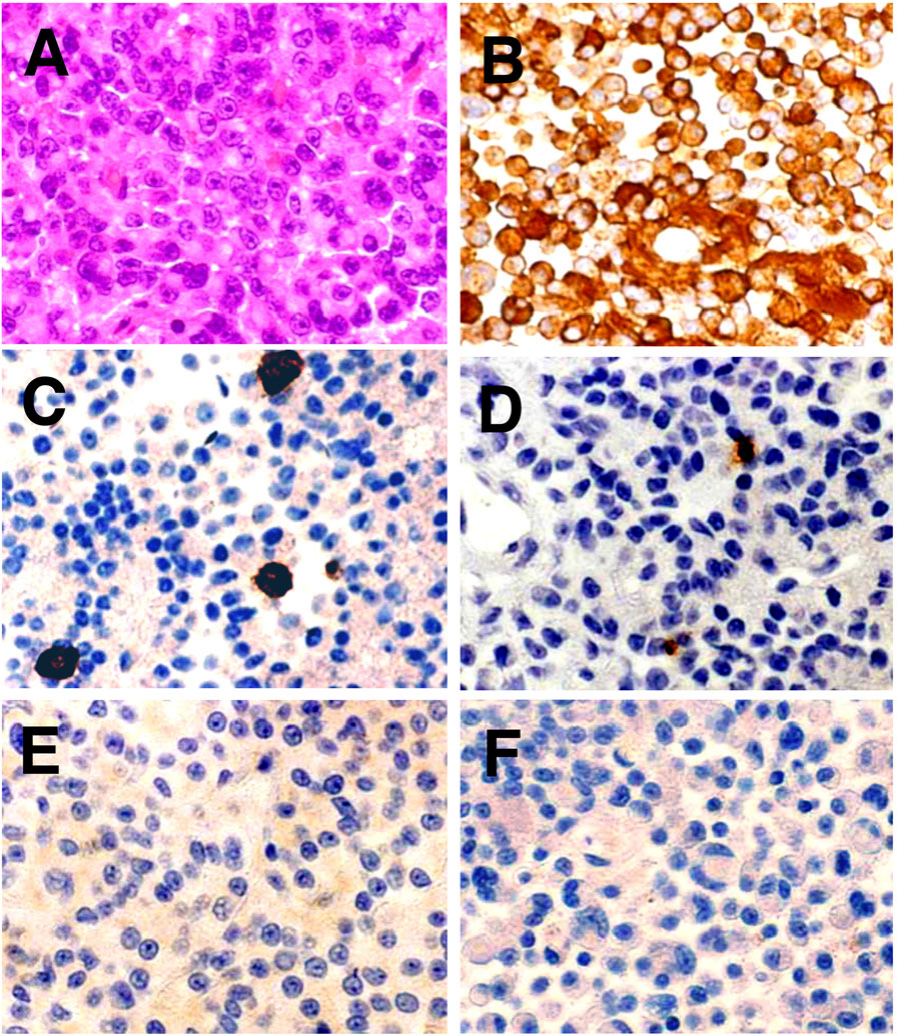
Histopathological and immunohistochemical study. Photomicrographs showing histopathological features and immunoprofile of a pituitary adenoma. Tissue prepared with H & E showing solid tumor nest (**a**). Immunohistochemical staining of the tumor for GH (**b**), PRL (**c**), ACTH (**d**), LH (**e**), and FSH (**f**). Immunohistochemically, the tumor cells show diffuse cytoplasmic reactivity to anti-GH antibody and focal reactivity to anti-ACTH. Original magnification × 100

Pathological examinations showed that hematoxylin-Eosin staining of the resected tumor sample revealed monotonous multiplication of acidophilic small round cells, mainly in a well-developed small blood vessel, which was in accordance with the diagnosis of pituitary adenoma (Fig. 2a). On immunohistochemical staining, most of the tumor cells were strongly stained by GH antibody, while some cells showed positive staining for PRL. Also, some tumor cells were strongly positive for ACTH but sparse (Fig. 2b-f), similarly to a previous report [4].

The patient was discharged without any post-surgical complications. However, because the postoperative basal plasma GH and IGF-1 levels were not normalized, another examination was performed 2 months after the surgery. Contrast MRI showed that the pituitary adenoma was resected, but it was difficult to evaluate the residual lesion in detail. To exclude the effect of hyperglycemia on hormones, a 75-g OGTT test was conducted after glycemic control was stabilized. The GH level was reduced to one-tenth or lower than that before the surgery, but it stayed at 10 ng/dL in the presence of elevated blood glucose levels, which did not meet the criteria for remission. In addition, the paradoxical response of GH to LHRH and TRH loadings was improved but remained. Thus, OCT-LAR (10 mg/mo) administration was started as posttreatment medication under non-remission status. Because the GH and IGF-1 control were insufficient, the octreotide dose was increased, with careful monitoring for blood glucose level and insulin secretion. Then, gradual decreases in GH and IGF-1 levels were observed. Considering a potential reoperation, we carefully monitored the patient response to the octreotide treatment.

Regarding the HPA axis, while the levels of GH and IGF-1 decreased with octreotide administration, ACTH level was still high and cortisol was not suppressed by DEX loading test, suggesting the possibility that ACTH-producing cells were in the residual tumor (Table 1). In addition, the ACTH level was not associated with blood glucose control or the reduction of the GH and IGF-1 levels.

### Immunofluorescence histochemistry of primary cultured cells

Cultured cells were transferred to 8-well PLL-coated slide-chamber. After 48 h incubation, they were fixed with methanol and were blocked with gout serum. After blocking, the ACTH and Pit-1 protein was detected by incubation with anti-ACTH (2F6, sc-69,648, Santa Cruz Biotechnology, Dallas, USA), anti-Pit-1 antibody (X-7, sc-442, Santa Cruz) at 4 °C overnight. Bound primary antibodies were detected by incubation with Alexa 488. Immunofluorescent signals were observed by confocal microscope (Zeiss, Germany).

### Immunofluorescence histochemistry of pituitary-tumor tissue

Paraffin embedded tissues were sectioned at 10 μm. After deparaffinized and unmasking step, the ACTH and GH protein was detected by incubation with anti-ACTH、anti-GH antibody (A0570, Dako, Calfornia, USA) at 4 °C overnight. Bound primary antibodies were detected by incubation with Alexa 584 and 488 respectively. Immunofluorescent signals were observed by confocal microscope (Zeiss).

### RNA extraction and real-time RT-PCR

Total RNA was isolated from tissues of the pituitary tumor using the RNeasy Minikit (QIAGEN, Valencia, CA) according to manufacturer’s instructions. RNA was reverse transcribed by using SuperScript III First-Strand Synthesis System (Invitrogen, Carlsbad, CA). Quantitative PCR was performed using an ABI PRISM 7500 real-time PCR system (Applied Biosystems, Foster City, CA). Quantitative PCR was conducted using primers for *GH*, *Pit-1*, *POMC*, *Tpit*, and *NeuroD1*, as noted in Table 2.

**Table 2:** Oligonucleotide primers for human *GH, Pit-1, POMC, Tpit, and NeuroD1*

*GH* forward*,* 5′-TCAAGCAGACCTACAGCAAG-3′	
*GH* reverse, 5′-ACCTTGTCCATGTCCTTCCT-3′	
*Pit-1* forward, 5′-TCTCCAACCATGCCACCAAT-3′	
*Pit-1* reverse, 5′-TGCCATCACTCCATAGGTTG-3′	
*POMC* forward, 5′- GCCTGGAAGATGCCGAGAT −3′	
*POMC* reverse, 5′- TGCTTTCCGTGGTGAGGTC −3	
*Tpit* forward, 5′- GCAAAGTGAAGCTGACCAAC-3′	
*Tpit* reverse, 5′- GCACTTCCAACACGCACTAT −3′	
*NeuroD1* forward, 5′- AAGAGGAAGAGGAGGATGAC −3′	
*NeuroD1* reverse, 5′- CGTTAGCCTTCATGCGTCTCAA −3’	

### Cell culture

Dissected pituitary tumor was incubated with collagease 1 mg/ml for 60 min at 37 °C. Extracted cells were filtered through nylon filter (100 μm), then they were centrifuged by 1500 rpm for 5 min. Pellet was suspended with condition medium (Dulbecco’s Modified Eagle Medium, penicillin (100 U/ml), streptomycin sulfate (100 μg/ml; Invitrogen), and 10% fetal bovine serum). Cells were plated in a PLL-coated 6-well plate in condition medium.

### Quantitative PCR analysis of anterior-pituitary related-genes

**Fig. 3:**
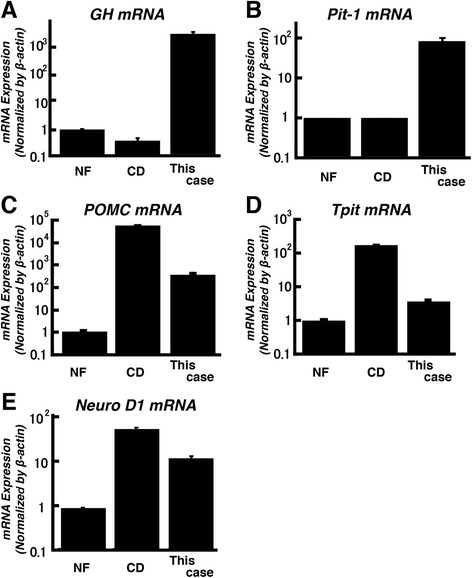
Evaluation of mRNA expression by real-time qPCR analysis. Total RNA was extracted from the surgically removed tumor and in vitro analysis using the tumor demonstrated that mRNA of *GH* (**a**), *Pit-1* (**b**), *POMC* (**c**), *Tpit* (**d**)*,* and *Neuro D1* (**e**) were expressed more in the tumor than a nonfunctioning pituitary tumor, suggesting that the tumor was able to secrete both ACTH and GH. Data represent mean ± S.E.M

In vitro analysis was performed to analyze hormone production ability (GH and ACTH) beyond the lineage in the pituitary tumor. Each surgical obtained pituitary-tumor samples, whose patients diagnosed with non-functioning and Cushing disease respectively, showed null cell type and ACTH-positive densely-granulated type pathologically, which were consistent with non-functioning and ACTH-producing adenoma respectively. These samples were used for comparison. First, total RNA of the resected tumor tissue was extracted. A quantitative gene expression analysis was performed using the real-time quantitative polymerase chain reaction method. *GH* and *Pit-1* mRNA showed higher expression in the tumor tissue than nonfunctional adenoma (NFA) or Cushing’s disease (CD) (Fig. 3a and b). Moreover, *POMC, Tpit,* and *NeuroD1* mRNA showed higher expression in the tumor tissue than NFA, but lower expression than CD, suggesting that expression of 2 types of transcriptional factors might be associated with clinical manifestation (Fig. 3c-e).

### Double fluorescent immunostaining analysis of the cells from the tumor tissue and primary culture

**Fig. 4:**
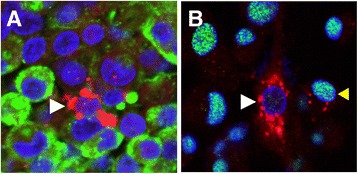
Immunofluorescent analysis using (**a**) tumor tissue and (**b**) its primary culture. (**a**) ACTH-stained (*red*) cells existed in the majority of GH-stained (*green*) cells. *White arrow* indicates an ACTH-stained cell (× 400). (**b**) The expression of Pit-1 (green) decreased in the nuclei of the cells with their cytosols stained by ACTH compared to GH-stained cells. *White arrow* indicates an ACTH-stained cell in the cytoplasm. *Yellow arrow* indicates a Pit-1-stained cell in the nucleus (× 400)

An immunohistological study was performed using a paraffin tissue sample and cell staining from the primary culture of the neoplastic cells derived from the resected tumor tissue. Double immunostaining for ACTH and GH demonstrated coexistence of ACTH-positive cells (GH-negative) among the GH-positive tumor cells in the tumor (Fig. 4a). Additionally, Pit-1 expression was reduced in the ACTH-positive from the tumor tissue primary culture (Fig. 4b). Considering the fact that Tpit determines alternate fates during pituitary cell differentiation, Tpit would be involved in the pathophysiology of ACTH positive cells in this tumor of presented case [11]. These results suggested a heterogeneous mixture of 2 types of cells with different functional lineages in the tumor (bimorphous), indicating that these different cells in the tumor secreted GH and ACTH autonomically.

## Discussion and Conclusions

Anterior-pituitary cells are generally classified into three different functional lineages according to the expression pattern of specific transcription factors as follows: POMC/ACTH, FSH-LH, and GH-PRL-TSH. Pituitary homeobox 1 (Ptx1) is a transcription factor that is universally present in the anterior-pituitary cells [5, 12]. Ptx1 is also expressed in pituitary adenoma beyond functional lineages, and it functions synergistically with NeuroD1/2, SF-1/Ad4BP, and Pit-1, increasing the expression levels of POMC/ACTH, FSH-LH, and GH-PRL-TSH, respectively. Therefore, concomitant production of PRL and TSH, which belong to the Pit-1 lineage, is often accompanied by GH-producing pituitary adenomas as the same lineage. However, it is relatively uncommon that GHoma produces POMC/ACTH because they are different lineages. Nine cases of concomitant ACTH- and GH-producing tumor have been reported thus far, including the present case (Table 3) [4, 6, 7, 13–17]. Each case was examined through electron microscope images or immunohistological studies; among them, 5 cases involved a mixture of 2 types of tumor cells (bimorphous), and in 1 case, 2 types of hormones were produced from the same cells.

**Table 3:** Summary of past reports related to GH and ACTH double secretion pituitary tumor

Reference	Case	GH	ACTH	Analysis	Cause of ACTH and GH hypersecretion
1991 Arita et al. [6]	29 y.o. female	GHoma	SCD	Electron microscope	Bimorphous tumor (heterogeneous)
1994 R. L. Apel et al. [13]	76 y.o. female	GHoma	SCA	Electron microscope	Bimorphous tumor (heterogeneous)
1998 K. Kovacs et al. [14]	62 y.o. male	GHoma	N.D.	Electron microscope ISH	Bimorphous tumor (heterogeneous)
2001 N. Mazarakis et al. [15]	53 y.o. male	GHoma	SCA	IHC	ACTH: hyperplasia
2002 Kageyama et al. [4]	45 y.o. female	GHoma	CD	IHC	Bimorphous tumor (heterogeneous)
2002 Tahara et al. [7]	53 y.o. female	Subclinical GHoma	CD	IHC ISH	The same cell (double producer)
2006 Tsuchiya et al. [16]	54 y.o. male	GHoma	SCD	IHC	N.D.
2009 Oki et al. [17]	36 y.o. male	GHoma	SCD (HMW ACTH)	IHC	N.D.
Our case	45 y.o.male	GHoma	SCD	IHC in vitro	Bimorphous tumor (heterogeneous)

Abbreviations as follows: *Ghoma* GH producing adenoma, *SCD* Subclinical Cushing disease, *CD* Cushing disease, *SCA* silent corticotroph adenoma, *HMW* high molecular weight, *ISH* in situ hybridization, *IHC* immunohistochemistry, and *N.D.* not determined

In the present case, coexistence of ACTH-producing cells was confirmed in tumor tissue mainly comprising GH-producing cells by immunohistochemical staining in the resected tumor (Fig. 2b). The gene expression analysis of the pituitary differentiation-related transcription factor in the tumor tissue revealed a significant increase in *NeuroD1* mRNA expression level, an upstream transcriptional factor of POMC, accompanied by a strong *Pit-1* mRNA expression (Fig. 3b, d, and e). Thus, the aberrant expression of the transcription factor may be the cause of the bimorphous tumor. To clarify the correlation between GH and ACTH production ability at the single-cell level, immunohistochemical analysis showed reduced Pit-1 expression level and GH-negative staining in the ACTH-producing cells, suggesting that the tumor was bimorphous and composed of tumor cells with different functional lineages (Fig. 4). Nevertheless, the mechanism causing a mixture of cells with different transcription factors in a tumor is unknown. There are some possibilities as follows: (1) tumor cells with progenitor properties acquired 2 types of lineages, (2) a genetic transformation occurred from monoclonal GH-producing tumor cells, and (3) normal POMC-positive cells coexisted with tumor cells during tumor onset and the proliferation process. To confirm these hypotheses and examine the molecular mechanism of the pituitary tumor onset and acquisition of the functions, analysis of gene expression profiles at the single-cell level is necessary.

It is sometimes difficult to read HPA axis in acromegaly. It has been reported that GH and IGF-1 reduce the activity of 11β-HSD, an enzyme that metabolizes cortisone into cortisol and affects cortisol metabolism in the peripheral tissue [9, 18]. Excess secretion of GH and IGF-1 inhibits 11β-HSD-1 activation, resulting in reduced cortisol concentration in the peripheral tissue and increased ACTH expression. Thus, the 0.5-mg DEX suppression test, which is often used as a SCD-functional screening-test, does not elicit an adequate effect, which easily leads to a positive finding. However, it has been reported that a patient with Cushing’s disease was spared cushingoid features because of a defect in a peripheral conversion of cortisone to cortisol. If GH and IGF-1 reduce 11β-HSD activity, co-existence of Cushing disease cannot be avoided [19]. In addition, the HPA axis is activated with insulin resistance, which is also considered to cause reduced sensitivity to DEX suppression [8]. The effect of these mechanisms on increased ACTH and cortisol levels cannot be completely excluded in the present case. However, considering the urine cortisol of more than 100 μg/day (Table 1), the abnormal ACTH/cortisol levels even after blood glucose control was normalized, and the result of the in vitro analysis of mRNA expressions in tumor cells and double immunostaining (Figs. 3 and 4), we suggest that the main pathological cause was associated to 2 types of GH- and ACTH-producing cells that function autonomously in the tumor. To deeply evaluate pathology of GHoma with SCD-because GH, IGF-1, and insulin resistance influence cortisol metabolism-it is important to add cases and perform a careful examination of hormonal dynamics before and after glucose, GH, and IGF-1 levels are improved.

This is the first report of immunohistochemistry analysis of tumor-derived primary culture cells and quantitative-expression analyses of transcriptional factors of the tumor tissue for a case of GHoma with SCD. In the present case, double immunostaining and gene expression analysis suggested that the multihormonal pituitary adenoma concomitant with Pit-1 and Tpit lineage cells caused acromegaly associated with SCD.

## Abbreviations

ACTH: Adrenocorticotropic hormone
BS: Blood sugar
CRH: Corticotropin-releasing hormone
FSH: Follicle-stimulating hormone
GH: Growth hormone
IGF-1: Insulin-like growth factor-1
LH: Luteinizing hormone
MRI: Magnetic resonance imaging
PRL: Prolactin
SD: Standard deviation
TSH: Thyroid-stimulating hormone
